# Intra-articular delivery of micronized dehydrated human amnion/chorion membrane reduces degenerative changes after onset of post-traumatic osteoarthritis

**DOI:** 10.3389/fbioe.2023.1224141

**Published:** 2023-09-06

**Authors:** Angela S. P. Lin, David S. Reece, Tanushree Thote, Sanjay Sridaran, Hazel Y. Stevens, Nick J. Willett, Robert E. Guldberg

**Affiliations:** ^1^ Phil and Penny Knight Campus for Accelerating Scientific Impact, University of Oregon, Eugene, OR, United States; ^2^ Wallace H. Coulter Department of Biomedical Engineering, Parker H. Petit Institute for Bioengineering and Bioscience, Georgia Institute of Technology, Atlanta, GA, United States

**Keywords:** osteoarthritis, amniotic membrane, intra-articular, delayed treatment, medial meniscal transection, 3D EPIC-μCT

## Abstract

**Background:** Micronized dehydrated human amnion/chorion membrane (mdHACM) has reduced short term post-traumatic osteoarthritis (PTOA) progression in rats when delivered 24 h after medial meniscal transection (MMT) and is being investigated for clinical use as a disease modifying therapy. Much remains to be assessed, including its potential for longer-term therapeutic benefit and treatment effects after onset of joint degeneration.

**Objectives:** Characterize longer-term effects of acute treatment with mdHACM and determine whether treatment administered to joints with established PTOA could slow or reverse degeneration. Hypotheses: Acute treatment effects will be sustained for 6 weeks, and delivery of mdHACM after onset of joint degeneration will attenuate structural osteoarthritic changes.

**Methods:** Rats underwent MMT or sham surgery (left leg). mdHACM was delivered intra-articularly 24 h or 3 weeks post-surgery (*n* = 5–7 per group). Six weeks post-surgery, animals were euthanized and left tibiae scanned using equilibrium partitioning of an ionic contrast agent microcomputed tomography (EPIC-µCT) to structurally quantify joint degeneration. Histology was performed to examine tibial plateau cartilage.

**Results:** Quantitative 3D µCT showed that cartilage structural metrics (thickness, X-ray attenuation, surface roughness, exposed bone area) for delayed mdHACM treatment limbs were significantly improved over saline treatment and not significantly different from shams. Subchondral bone mineral density and thickness for the delayed treatment group were significantly improved over acute treated, and subchondral bone thickness was not significantly different from sham. Marginal osteophyte degenerative changes were decreased with delayed mdHACM treatment compared to saline. Acute treatment (24 h post-surgery) did not reduce longer-term joint tissue degeneration compared to saline. Histology supported µCT findings and further revealed that while delayed treatment reduced cartilage damage, chondrocytes displayed qualitatively different morphologies and density compared to sham.

**Conclusion:** This study provides insight into effects of intra-articular delivery timing relative to PTOA progression and the duration of therapeutic benefit of mdHACM. Results suggest that mdHACM injection into already osteoarthritic joints can improve joint health, but a single, acute mdHACM injection post-injury does not prevent long term osteoarthritis associated with meniscal instability. Further work is needed to fully characterize the durability of therapeutic benefit in stable osteoarthritic joints and the effects of repeated injections.

## Introduction

Osteoarthritis (OA) is a progressive, degenerative joint disease that is a leading cause of chronic disability and pain in the US, estimated to affect over 13% of the US adult population (Cisternas et al., 2016). There are no approved disease modifying or regenerative therapies to alter degenerative processes or eliminate the need for eventual total joint replacement. OA is a complex disease characterized by morphological and compositional changes in joint tissues including cartilage surface erosion, loss of proteoglycans, lesion formation, osteophyte formation, and synovial inflammation (Hunter, 2011). Growing evidence indicates that OA has diverse pathophysiology and etiology, meaning there are different origins and various ways to categorize subpopulations, and a patient’s specific presentation of localized tissue degeneration, OA phenotype, and other biological and environmental factors may impact the efficacy of a therapeutic for that individual (Mobasheri and Batt, 2016; Roze et al., 2016; Salazar-Noratto et al., 2019a; Van Spil et al., 2019). Current standards of therapy only provide generalized symptomatic relief in the form of non-steroidal anti-inflammatory drugs, steroid injections, analgesics, and viscosupplementation (Clouet et al., 2009). A number of candidate drugs targeting disease modification are being evaluated in clinical trials, but none have clearly demonstrated modified disease progression for any OA phenotypes to date (Roze et al., 2016; Van Spil et al., 2019; Deveza et al., 2017; Ghouri and Conaghan, 2019; Karsdal et al., 2016; Martel-Pelletier et al., 2012; Waarsing et al., 2015; Wieland et al., 2005). These developments in OA characterization and treatments point toward the need for greater understanding of localized OA phenotypic changes and more complete assessments of how existing and new therapies affect different joint tissues and OA subtypes.

This study focuses on an extracellular matrix (ECM)-based approach using a placentally-derived amnion/chorion membrane material, which has been shown in other applications to possess anti-inflammatory properties, display low immunogenicity, and promote wound healing while inhibiting scar formation (Faulk et al., 1980; Hannon et al., 2019; Hao et al., 2000). The product utilized in this study is considered a human cellular and tissue-based product (HCT/P) by the U.S. Food and Drug Administration (FDA), and this umbrella category includes stem cell and ECM-based approaches. The regulatory processes covering these products range from clinicians being allowed to use them with no requirements to be licensed, approved, or cleared by FDA to being required to undergo premarket review of safety and efficacy data and postmarket reporting activities (GovInfo, 2019; FDA, 2007; FDA, 2017). The guidelines for and sub-categorization of developed products have evolved over time, which also means that some products may have shifted from being less to more regulated. This has encouraged a more systematic evidence-based approach towards translation that encourages collaboration between researchers, regulators, and industry to provide patients with safe products that have more clearly defined mechanisms of action, evidence to support use, and guidelines on treatment timing (Jones et al., 2019a; Jones et al., 2019b; Lamplot et al., 2020; LaPrade et al., 2016; Rodeo, 2018). Even for products that are already undergoing clinical trials, evolutions in regulatory oversight have put a higher value on researchers to continue elucidating effects of candidate biologics preclinically in order to identify which products may have higher efficacy and specificity for different joint tissues in various OA phenotypes as well as the appropriate timing for which therapeutics need to be administered (Hannon et al., 2019; Lamplot et al., 2020; Rodeo, 2018).

Several clinical case series have recently been published in support of the use of particulate amniotic membrane ECM-based materials (amnion, chorion, umbilical cord, amniotic fluid) to treat OA (Mead and Mead, 2020; Natali et al., 2022; Alden et al., 2021; Bennett, 2019; Castellanos and Tighe, 2019; Farr et al., 2019; Vines et al., 2016). Four of the studies specifically addressed knee OA, though they were relatively small preliminary studies (6, 20, 25, and 42 qualifying patients), and indicated that single injections with suspensions of either amniotic membrane, amniotic membrane combined with umbilical cord, or amniotic membrane combined with amniotic fluid may help reduce knee pain and improve function in moderate knee OA in the short term (Mead and Mead, 2020; Natali et al., 2022; Castellanos and Tighe, 2019; Vines et al., 2016). One large retrospective case series has been published on the use of injectable micronized dehydrated human amnion/chorion membrane (dHACM) alone, and it included review of 100 knees treated with the product. Patient-reported outcome scores (including pain, daily living, sports/recreation, and quality of life categories increased over time up to 6 months (Alden et al., 2021). One multi-center level I randomized controlled trial that enrolled 200 knee OA patients concluded that treatment with single injection of an amniotic membrane particulate/amniotic fluid suspension was superior to hyaluronic acid and saline at 3 and 6 months in terms of pain relief and improved function. Notably, significant improvements in patient-reported outcome categories of pain and daily living at 3 and 6 months and sports/recreation and quality of life at 6 months were shown (Farr et al., 2019). At the time of writing, there were about 20 clinical trials of an interventional nature for osteoarthritis using amniotic materials listed at clinicaltrials.gov: Four had been withdrawn, two were not yet recruiting, five were recruiting, and eight were completed, but only one of the completed studies had results included on clinicaltrials.gov. Most of the clinical studies have been completed or are in progress, suggesting that those products are likely still viable candidates for OA disease modifying therapies. However, the limited available clinical trial results and number of withdrawn trials further highlight that variability in efficacy exists. This supports the continuing need for preclinical studies, such as this one, to improve scientific understanding of the effects being produced by such products.

In a previous study, we demonstrated the therapeutic potential of an injectable, micronized dHACM product (mdHACM; EpiFix^®^ Injectable, MiMedx Group, Inc. Marietta, GA) to slow post-traumatic joint degeneration by testing its efficacy in a short-term preclinical rat post-traumatic OA model. A single intra-articular injection of mdHACM administered 24 h after surgical injury inhibited lesion formation and reduced cartilage sulfated glycosaminoglycan (sGAG) loss in the widely used rat medial meniscal transection (MMT) model at 3 weeks post-injury (Willett et al., 2014). 3D cartilage morphology and composition metrics were quantified using equilibrium partitioning of an ionic contrast agent with microcomputed tomography (EPIC-µCT). Results demonstrated ameliorated OA development at 3 weeks post-surgery with immediate delivery of mdHACM treatment, but critical issues such as the duration of therapeutic effect and the potential to treat joints with established OA were not evaluated.

In the present study, we evaluated both the longer-term effects (6 weeks post-surgery) of an acute (24 h) treatment of mdHACM and effects of delayed delivery, where mdHACM was injected 3 weeks post-MMT surgery when joint tissue degeneration has already begun. These conditions simulate two scenarios: (a) treatment immediately following acute injury that is known to induce post-traumatic OA and (b) treatment after symptoms have developed, which would be more consistent with a typical presentation of post-traumatic OA where the patient seeks medical care after becoming symptomatic.

The objectives of this study were to characterize the longer-term effects of immediate delivery (24 h post-surgery) of a micronized amnion ECM treatment and to determine whether this treatment could reduce the progression of cartilaginous and bony changes as evaluated by EPIC-µCT and histology. The hypotheses were that the duration of therapeutic effect of acute micronized amnion ECM treatment would extend to a 6 week endpoint and that delayed injection administered 3 weeks after induction of OA would attenuate further degenerative changes in cartilage and bone tissues in the rat MMT model.

## Materials and methods

### Preparation of mdHACM

mdHACM was manufactured using the proprietary PURION^®^ process and is compliant with the American Association of Tissue Banks’ regulations for donor tissues (MiMedx Group, Inc. Marietta, GA). This process produces a dehydrated, devitalized amnion and chorion tissue graft which is then sterilized and micronized.

### Surgical methods–Medial meniscal transection

Weight matched adult male Lewis rats (Charles River, Wilmington, MA) weighing 300–325 g, were acclimated for 1 week after arrival, housed throughout the study at a 12 h light/dark cycle. Animals were dual-housed except immediately after surgeries when they were single-housed until wound clips were removed (10–14 days post-surgery). Animals were anesthetized with isoflurane, given subcutaneous sustained-release buprenorphine (ZooPharm) for analgesia, and the skin over the medial aspect of the left femoro-tibial joint was shaved and aseptically prepared. A skin incision was made by scalpel, 1–1.5 cm in length on the medial aspect of the knee joint. The medial collateral ligament was exposed by blunt dissection of the muscle and transected with microdissection scissors to access the joint space starting from the distal side. Clearance on tibial and femoral sides of the meniscus was carefully established, facilitated by external rotation of the limb and clearing of surrounding connective tissues, and a full thickness cut was made through the full thickness of the meniscus to ensure destabilization (Janusz et al., 2002). For sham surgeries (*n* = 7), the medial collateral ligament was exposed and transected to visualize the meniscus and joint spaces, but the meniscus was not transected. The muscle was closed with 4.0 Vicryl sutures and the skin stapled using wound clips.

MMT animals received an intra-articular injection to the left knee at 24 h (acute treatment; *n* = 7) or 3 weeks (delayed treatment; *n* = 5) post-surgery of mdHACM (EpiFix^®^ Injectable, MiMedx Group Inc. Marietta GA). After resuspending mdHACM in 1 mL saline (80 mg/mL concentration), 50 µL solution was injected into the articular space (25 g needle, BD, Franklin Lakes, NJ). As a control, separate MMT animals received intra-articular injections of 50 µL saline at 24 h post-surgery (*n* = 6). All animals were euthanized at 6 weeks post-surgery via CO_2_ inhalation. The Georgia Institute of Technology IACUC approved all experimental procedures for these *in vivo* studies (Protocol #A12018).

### EPIC-µCT analysis of articular cartilage, subchondral bone, and osteophytes

Cartilage and subchondral bone morphology and composition were assessed using EPIC-µCT in the medial third of the medial tibial plateau as described previously (Thote et al., 2013; Willett et al., 2016; Xie et al., 2010; Xie et al., 2012; Xie et al., 2009). Dissected tibiae were immersion fixed in 10% neutral buffered formalin for 48 h then stored in 70% ethanol (v/v) until ready for scanning. Harvested hindlimbs were carefully microdissected to separate femora from tibiae, and in the process full transections of medial menisci were visually confirmed for each limb that received MMT surgery with intact menisci verified for sham surgeries. Immediately prior to scanning, tibiae were patted dry and then immersed in 2 mL of 30% Hexabrix™ 320 anionic contrast agent (Covidien, Hazelwood, MO) solution in phosphate buffered saline (without Ca^2+^ or Mg^2+^) at 37°C for 30 min (Xie et al., 2009; Palmer et al., 2006). Tibiae were removed from solution, patted dry, secured in sample holders with water at the bottom, sealed to maintain humid environment to prevent drying/shrinkage, and each scanned in the same orientation using a µCT40 (Scanco Medical, Brüttisellen, Switzerland). Scan and reconstruction settings: 45 kVp, 177 µA, 200 m integration time, isotropic voxel size of 16 µm, pixel matrix dimensions 1024 × 1024, scan time −26 min (Xie et al., 2009). Raw data were automatically reconstructed to axial 2D grayscale tomograms and orthogonally transposed to coronal and sagittal sections. Scanco evaluation software was used to assess local 3D morphologic and compositional measures, using semi-automated spatial segmentation and global thresholding parameters (expressed throughout the manuscript in mineral density units, mg hydroxyapatite (HA)/cm^3^), for the following OA-affected tissue types and to output slice images for further image processing.

**FIGURE 1:**
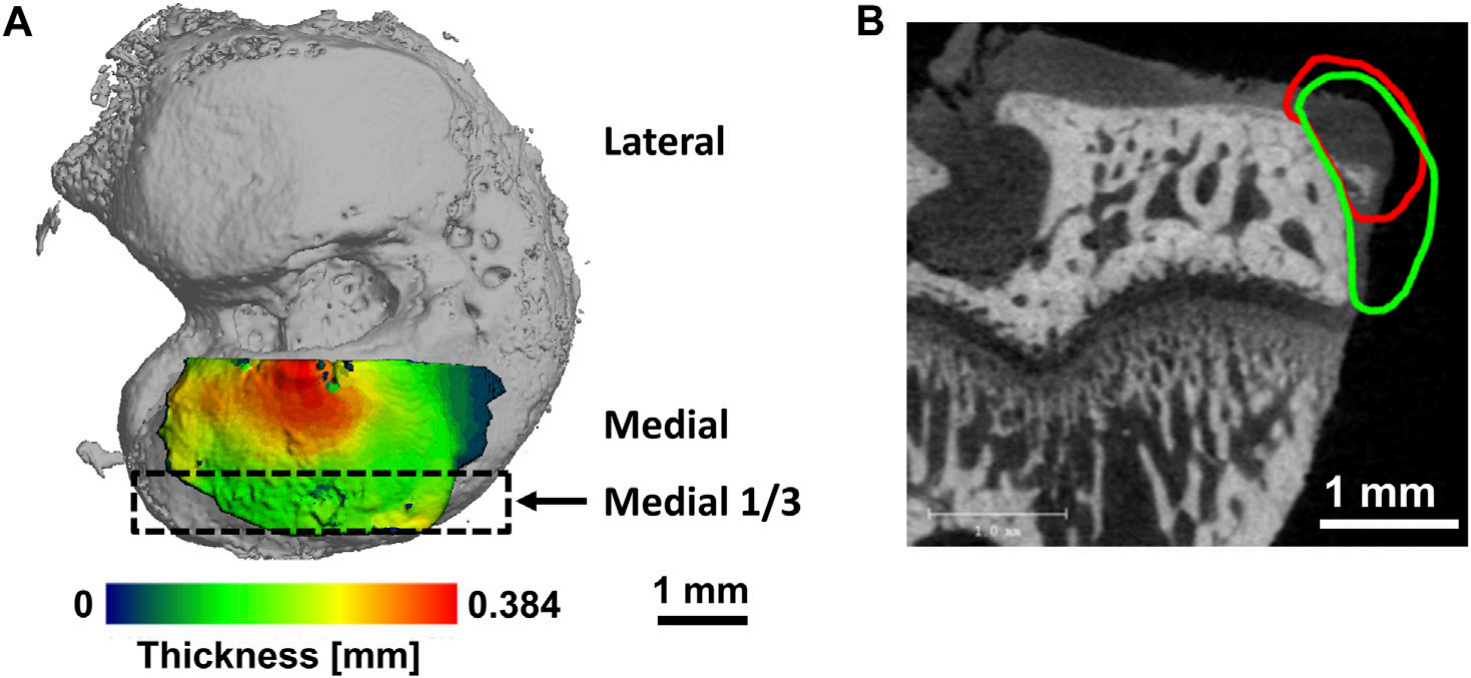
Representative figures illustrating evaluation areas. **(A)** Volumes of interest for cartilage on tibial plateau. Medial third of medial tibial plateau indicated by black rectangle. **(B)** Representative image of an osteophyte on the medial tibial margin in the rat medial meniscal tear model with contour for cartilage volume (red) and mineralized volume (green).

Articular cartilage: In sagittal sections, cartilage was contoured and thresholded with global segmentation parameters (Gauss sigma 1.0, support 1, lower and upper thresholds 179 and 740 mg HA/cm^3^). Images regionally evaluated in the medial third of the medial tibial plateau (Figure 1A). Direct distance transformation algorithms were used to quantify 3D cartilage thickness measures (Xie et al., 2009; Hildebrand and Rüegsegger, 1997; Laib et al., 2000; Lin et al., 2015). Consistent volumes of cartilaginous tissue excluding margins were annotated for evaluation based on image processing standards previously established (Reece et al., 2018). For regions surrounding lesions on the medial tibial plateau in this model and at this timepoint, previous studies have shown that the remaining cartilage tissue swells, which results in higher average thickness measured by contrast enhanced µCT (Willett et al., 2014; Thote et al., 2013; Willett et al., 2016; Lin et al., 2015; Reece et al., 2018; Reece et al., 2020). Average X-ray attenuation of contrast enhanced cartilage (expressed in units of mg HA/cm^3^) was also quantified and has previously been demonstrated to be inversely proportional to sulfated glycosaminoglycan (sGAG) content (Xie et al., 2010; Palmer et al., 2006). Degraded cartilage has a lower sGAG content and therefore, after equilibration in Hexabrix™, possesses higher contrast agent content and higher attenuation values. For cartilage surface roughness measurements, sequential 2D grayscale images of sagittal slices of the medial third of the tibial plateau were imported into MATLAB^®^ (MathWorks, Natick, MA). A custom built algorithm, using a global threshold to separate the bone and cartilage surface pixels from each other and the surrounding air pixels, was used to automatically scan each image sequentially to create a 3D digital representation of the cartilage and bone surfaces (Reece et al., 2018). The cartilage surface was fit with a 3D polynomial surface that was fourth order along the ventral/dorsal axis and second order along the medial/lateral axis. The surface roughness was then calculated as the root mean square of the differences between the cartilage and polynomial surfaces. Exposed subchondral bone was calculated as a measure of lesion formation by summing the total area where the bone and cartilage surfaces were separated by less than 2 pixels (32 µm).

Subchondral bone: Quantifications of subchondral bone, the layer of bone underlying articular cartilage, in the medial third of the medial tibial plateau were also performed via sagittal section images using Scanco evaluation software. This analysis region was directly adjacent to the cartilage evaluation region described above and indicated in Figure 1A. Subchondral bone mineral density and thickness were calculated after spatially isolating the bone using contouring and a threshold range of 740–3,047 mg HA/cm^3^.

Osteophyte: Defined as bony outgrowths with fibrocartilaginous tissue caps forming on the margins of articular weight bearing joints (van denBerg, 1999; van der Kraan and van den Berg, 2007) (Figure 1B), osteophytes in the medial tibial plateau were analyzed via coronal sections. Osteophyte cartilage volume was measured in volumes of interest that excluded peripheral soft tissue, and cartilage was segmented from air and bone using threshold range 179–670 mg HA/cm^3^. Osteophyte mineralized volume measurements were made in volumes of interest that did include the peripheral soft tissue, segmented from air, cartilage, and soft tissue using threshold range 670–3,047 mg HA/cm^3^.

### Histology

Following EPIC-µCT scanning, tibiae and femora were decalcified in Cal-Ex II (Thermo Fisher Scientific, Waltham, MA) on a shaker plate for 7–8 days. Dehydrated samples were routinely processed and embedded into paraffin blocks. Sections parallel to the sagittal plane were cut at 5 µm thickness and stained with hematoxylin and eosin (H&E).

### Statistical analysis

All quantitative data were expressed as mean + standard deviation (SD), with all data points displayed. All joint parameters between groups were evaluated using one factor (treatment) ANOVA with Tukey’s test for *post hoc* analysis except for the exposed bone parameter which was analyzed using the nonparametric Kruskal–Wallis one-way analysis of variance with Dunn’s *post hoc* analysis. Statistical significance was set at *p* < 0.05. All data were analyzed using GraphPad Prism software version 7.0 (GraphPad Software, Inc., La Jolla, CA).

## Results

**FIGURE 2:**
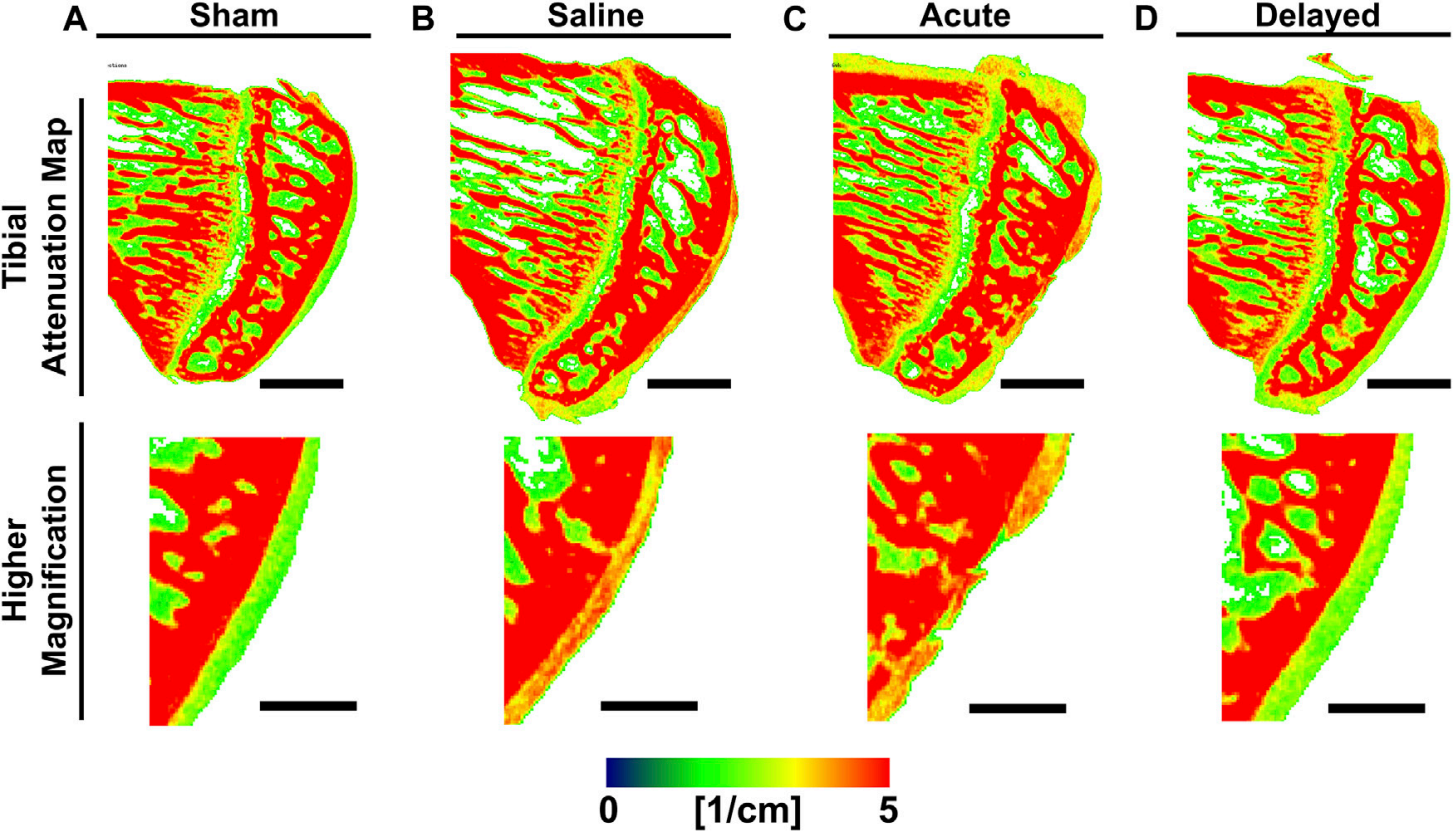
Representative medial tibial cartilage attenuation maps show lower attenuation in sham and delayed treatment samples. **(A)** Sham tibiae displayed smooth cartilage surface with green color indicating healthy sulfated glycosaminoglycan (sGAG) content. **(B)** Saline treated tibiae displayed high attenuation indicating lower sGAG content. **(C)** Tibiae receiving acute treatment (24 h post-surgery) of micronized dehydrated human amnion/chorion membrane (mdHACM) displayed high attenuation and initial lesion development. **(D)** Tibiae receiving delayed treatment (3 weeks post-surgery) of mdHACM displayed green central portion indicating healthy sGAG content. Pseudocolor X-ray attenuation bar (expressed in linear attenuation coefficient units [1/cm]): red = high attenuation/low sGAG content and green = low attenuation/high sGAG content. Scale bars = 1mm; higher magnification scale bars = 0.5 mm.

Representative sagittal sections from 3D contrast-enhanced cartilage X-ray attenuation maps showed qualitatively higher attenuation (i.e., lower sGAG content or more degenerated cartilage) at 6 weeks post-surgery for the saline and acute treatment groups compared to sham and delayed treatment groups (Figure 2, red = high attenuation/low sGAG content, green = low attenuation/high sGAG content).

**FIGURE 3:**
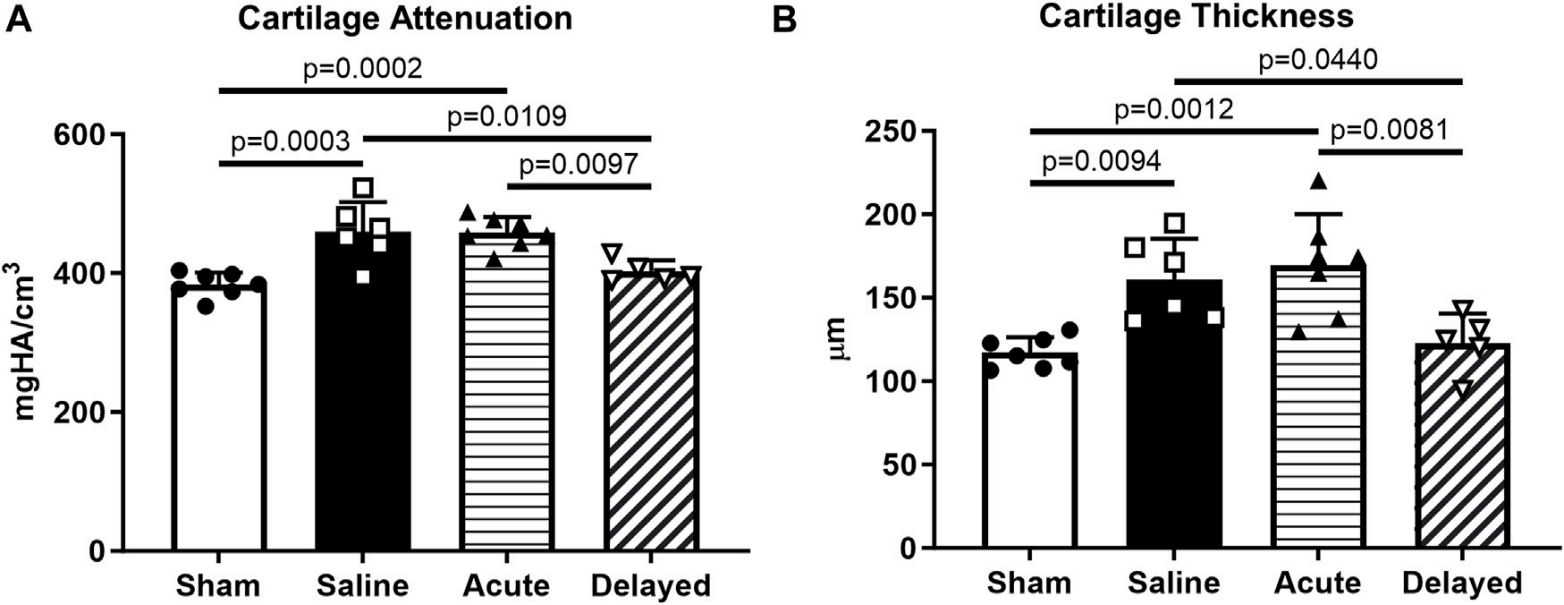
Delayed treatment (3 weeks post-surgery) of micronized dehydrated human amnion/chorion membrane (mdHACM) resulted in lower cartilage attenuation and thickness in the medial third of the medial tibial plateau compared to saline and acute mdHACM treatment (24 h post-surgery) groups. **(A)** Cartilage attenuation and **(B)** thickness were significantly higher than sham in the saline and acute mdHACM treatment groups, while both parameters in the delayed treatment group were significantly lower than saline and acute treatment groups. Data shown as mean + SD. Note that in this model and timepoint, degenerated cartilage has been shown to have higher X-ray attenuation (corresponding to lower sGAG content) and higher average thickness (more tissue swelling). n = 5-7.

Quantitative analysis of 3D EPIC-µCT images within the medial third of the medial tibial plateau revealed significantly lower cartilage X-ray attenuation in sham and delayed treatment groups compared to saline and acute treatment groups, indicating higher sGAG content in sham and delayed treatment groups (Figure 3A). Cartilage thickness was significantly lower in sham and delayed treatment groups compared to saline and acute treatment groups (Figure 3B). In contrast, no significant difference in cartilage attenuation or thickness was observed between the sham and delayed treatment groups.

**FIGURE 4:**
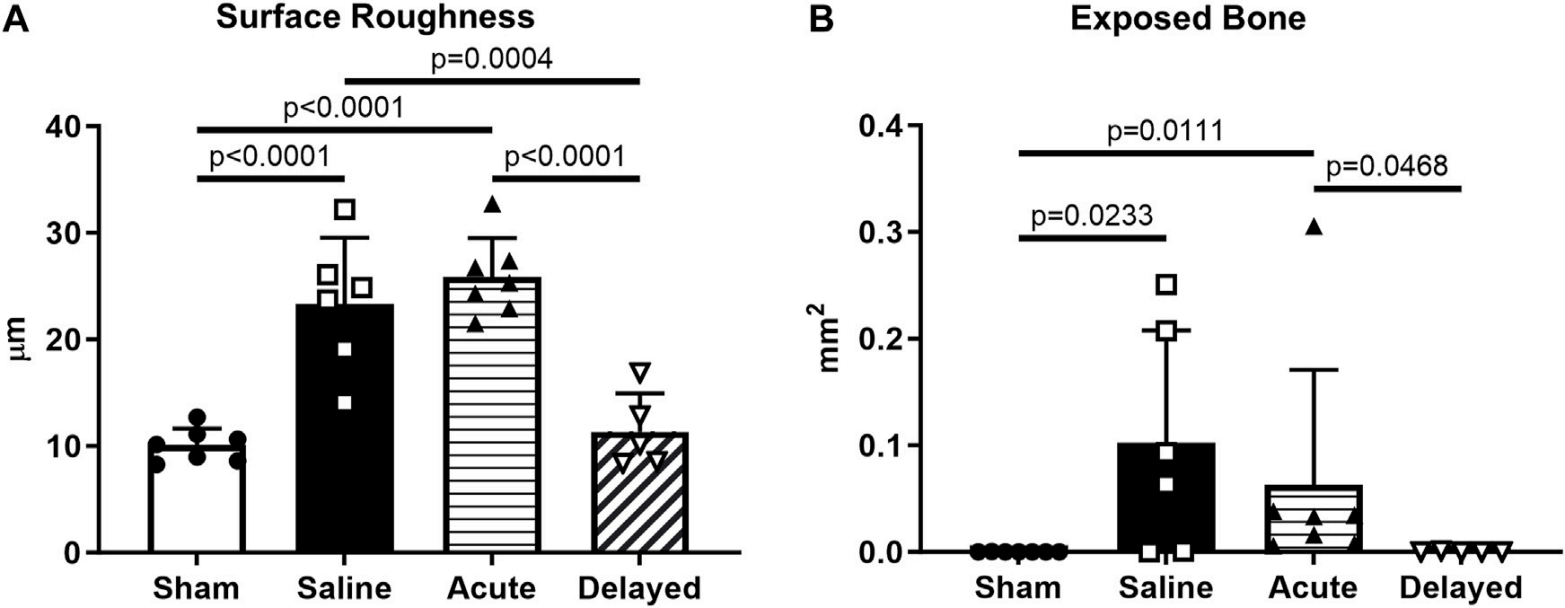
Articular cartilage surface roughness and exposed bone evaluated using custom program. **(A)** Cartilage surface roughness was significantly higher in the saline and acute (24 h post-surgery) micronized dehydrated human amnion/chorion membrane (mdHACM) treatment groups compared to sham, while surface roughness in the delayed mdHACM treatment (3 weeks post-surgery) group was significantly lower than saline and acute treatment groups. **(B)** Exposed bone/cartilage lesion area was significantly greater in saline and acute treatment groups compared to sham and significantly lower for delayed treatment compared to acute treatment. Data shown as mean + SD. n = 5-7.

Cartilage surface roughness and exposed bone area, additional 3D quantitative indicators of cartilage morphological change, were calculated using a custom MATLAB^®^ program developed and validated in previous work^60^. Analysis of medial third of the medial tibial plateau showed an increase in surface roughness in saline and acute treatment groups compared to sham and delayed treatment (Figure 4A). Exposed bone area was calculated as a measure of cartilage lesion size and was significantly higher in the saline and acute treatment groups compared to sham, and the delayed treatment group had significantly lower exposed bone area compared to the acute delivery group (Figure 4B).

**FIGURE 5:**
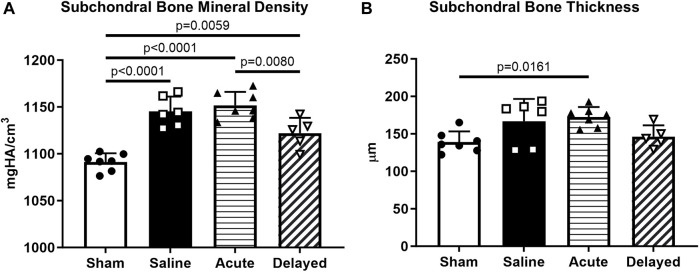
Subchondral bone quantifications. **(A)** Delayed treatment (3 weeks post-surgery) of micronized dehydrated human amnion/chorion membrane (mdHACM) resulted in lower subchondral bone mineral density in the medial third of the medial tibial plateau compared to acute mdHACM treatment (24 h post-surgery). Subchondral bone mineral density for the sham group was significantly lower than all other groups. **(B)** Acute dHACM treatment group demonstrated significantly greater subchondral bone thickness compared to sham. Data shown as mean + SD. n = 5-7.

Subchondral bone mineral density was significantly higher in saline, acute treatment, and delayed treatment groups compared to sham while the delayed treatment group had significantly lower mineral density compared to the acute treatment group (Figure 5A). Only the acute treatment group had significantly thicker subchondral bone compared to the sham group (Figure 5B).

**FIGURE 6:**
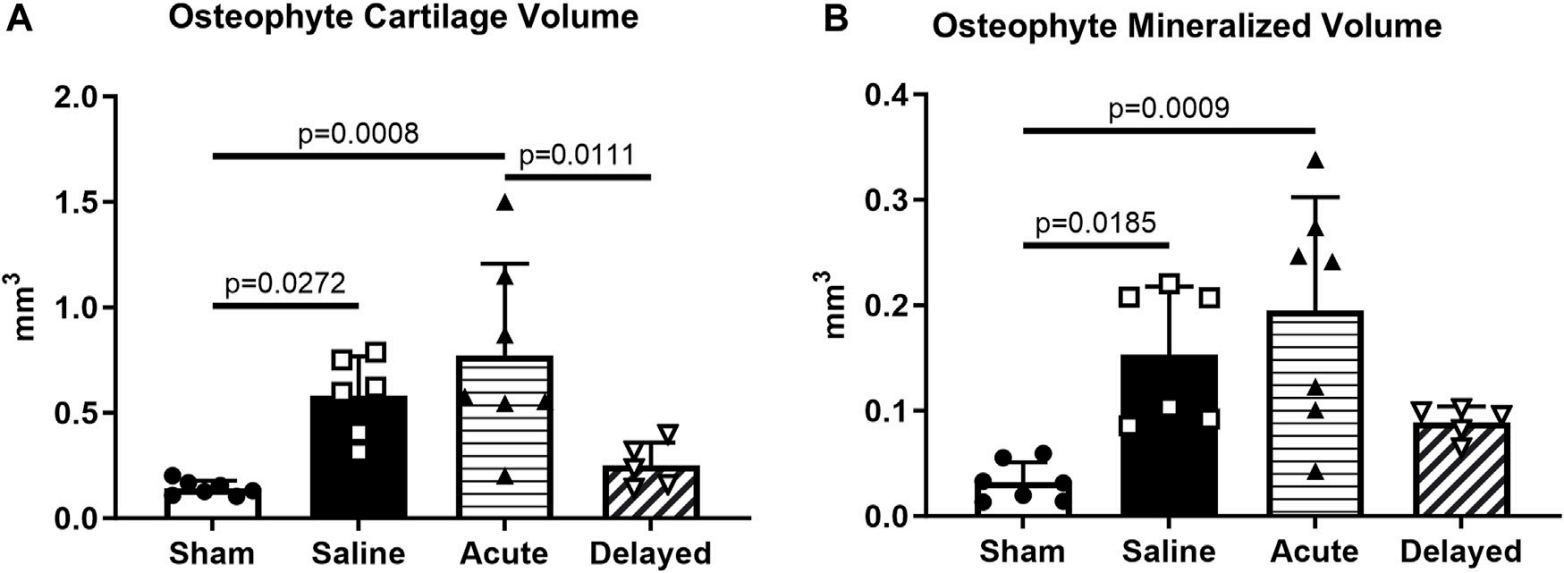
Saline and acute (24 h post-surgery) micronized dehydrated human amnion/chorion membrane (mdHACM) treatment groups display osteophyte progression compared to sham. **(A)** Osteophyte cartilage volume was significantly greater in saline and acute treatment groups compared to the sham group and significantly less in the delayed mdHACM treatment (3 weeks post-surgery) group compared to the acute treatment group. **(B)** Marginal mineralized volume was significantly greater in saline and acute treatment groups compared to the sham group. Data shown as mean + SD. n = 5-7.

Osteophyte cartilage volume was significantly higher in the saline and acute treatment groups compared to sham, and the delayed treatment group did not demonstrate significantly higher osteophyte cartilage volume compared to sham (Figure 6A). Similar results were observed for osteophyte mineralized volume with a significant increase for the saline and acute treatment groups compared to sham (Figure 6B). Osteophyte cartilage and mineralized volumes were not significantly increased in the delayed treatment group compared to the sham controls and osteophyte cartilage volume was significantly lower in the delayed treatment group compared to the acute treatment group.

**FIGURE 7:**
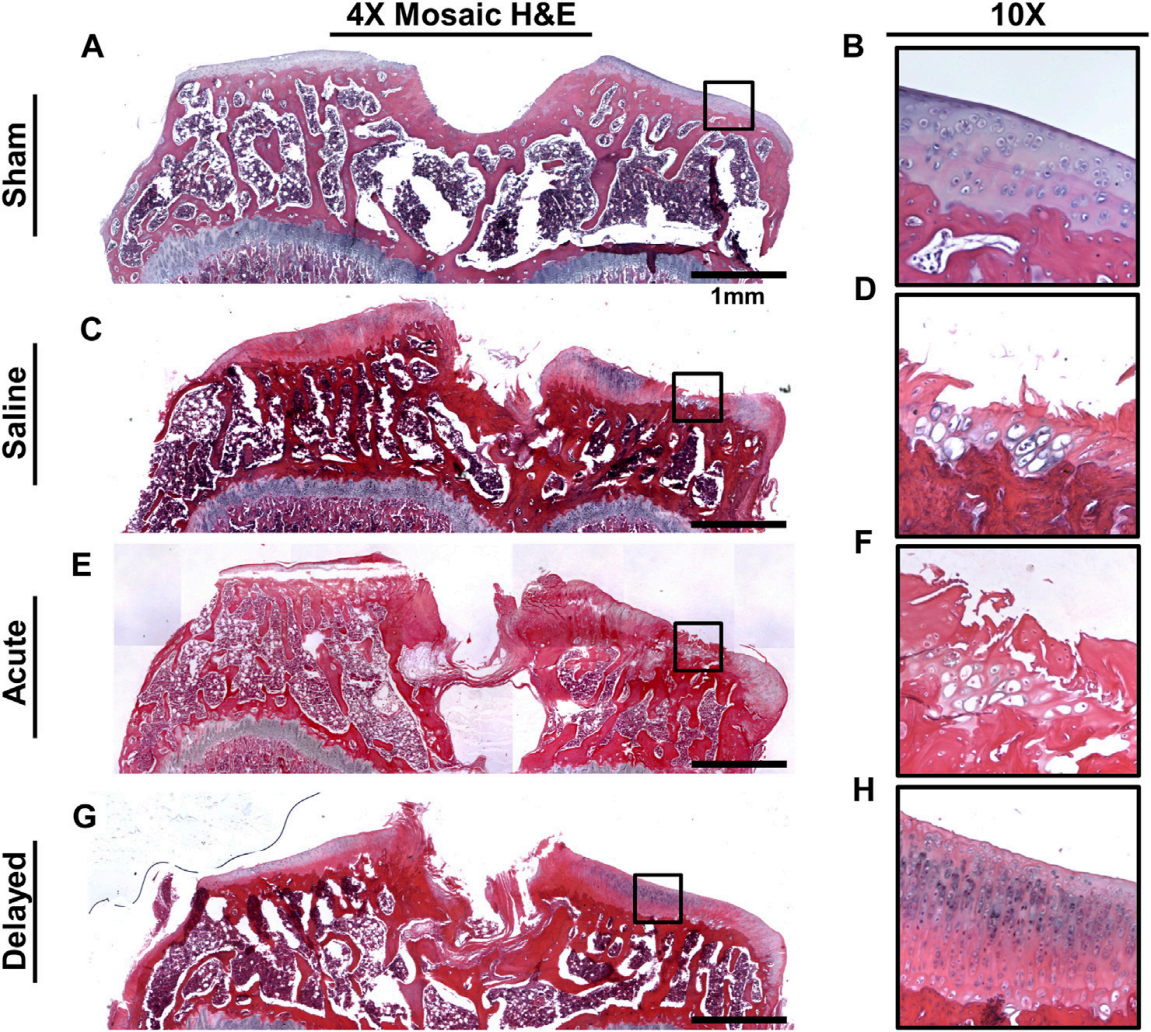
Representative H&E histology images. **(A, B)** Sham tibiae showed no cartilage damage and normal chondrocyte morphology. **(C, D)** Saline sample exhibited cartilage surface damage on the medial plateau indicated by fibrillations and lesions. **(E, F)** Acute micronized dehydrated human amnion/chorion membrane (mdHACM) treatment (24 h post-surgery) did not protect against cartilage damage as surface fibrillations were still observed. **(G, H)** Delayed mdHACM treatment provided some protection against cartilage damage as cartilage lesions and major fibrillations were not observed in this group. However, dense chondrocyte columns with elongated cell morphology and potential cloning chondrocytes were observed at 10× magnification in the delayed treatment group.

Histology was performed on tibiae and femora (with synovium attached) at the 6 week time point. For femora, previous studies have shown evidence of mdHACM in the synovium at a 3 week end point (Willett et al., 2014), but in this study no mdHACM was visible in the synovium surrounding the femur (images not shown). Representative images of coronal tibial sections showed degradation of cartilage surface in the saline (Figures 7C, D) and acute treatment group (Figures 7E, F). The sham group demonstrated smooth cartilage surfaces with normal chondrocyte morphology (Figures 7A, B). The delayed treatment group (Figures 7G, H) demonstrated no fibrillations and relatively smooth cartilage surfaces. However, cell morphology suggests the tissue has not been restored to completely healthy state as 10X images show densely packed chondrocytes in columnar structures with potential presence of cloning/clustering chondrocytes.

## Discussion

In this preclinical study, contrary to our hypothesis, the therapeutic benefit of a single intra-articular injection of mdHACM (from a single donor) at 24 h post-surgery was not sustained out to 6 weeks. Lack of protective effects of a single 24 h mdHACM injection may be a consequence of the unrepaired destabilization of the joint and subsequent advanced disease development throughout the 6 week study period. The acute treatment group in this study was intended to simulate therapeutic delivery directly after a traumatic injury known to produce downstream degenerative effects (e.g., clinically delivering a therapy immediately after a meniscus tear) and assess whether the single early injection prior to symptom onset could stave off degeneration. Delayed treatment was intended to be more representative of the typical clinical scenario, where a patient seeks treatment after disease symptoms have already developed. In contrast to acute treatment, a delayed intra-articular injection of mdHACM delivered after joint tissues had already demonstrated osteoarthritic changes resulted in reduced disease progression at the 6 week endpoint compared to saline controls, and for some parameters (such as medial third cartilage X-ray attenuation, thickness, surface roughness, exposed bone, subchondral bone thickness, and osteophyte metrics) there were no significant differences compared to sham joints. Our data suggest that mdHACM could provide protection against continued articular cartilage, subchondral bone, and osteophyte changes, but bony tissues may be protected to a lesser degree.

In this study, we chose to perform 3D contrast-enhanced µCT analysis over semi-quantitative histomorphometry of all samples and provided representative histological depictions of structural and compositional effects as supporting data. A number of previous studies have shown combinations of µCT and histopathology data and demonstrated that 3D µCT quantifications are equally if not more powerful for sensitively characterizing structural changes due to disease and treatment compared to 2D semi-quantitative scoring methods (Willett et al., 2014; Thote et al., 2013; Willett et al., 2016; Lin et al., 2015; Reece et al., 2018; Reece et al., 2020; Thote, 2014). One such study compared and correlated various contrast-enhanced µCT 3D metrics with 2D histopathology analyses and demonstrated that µCT analyses provide fully quantitative and highly sensitive measures of joint degeneration with lower sample numbers compared to histopathology (Willett et al., 2016). Additional advantages of µCT analyses include mitigating scoring variability or bias and providing volumetric data instead of sampling 2D sections from a 3D region of interest. However, histology provides valuable information about cellular and molecular changes within joint tissues that are not available through µCT analyses. One limitation in the current study due to prioritizing µCT analysis over semi-quantitative histomorphometry scoring was that while femoral histology sections were examined for presence of mdHACM in synovium (images not shown), changes in synovial membrane tissues due to disease and therapeutic delivery could not be accurately assessed with the microdissected tissues (typically performed on intact joint histology).

It is important to note that several ECM-based therapeutics are currently being investigated in clinical trials for treating OA. However, there are very limited available clinical trial results published, and these therapeutics have shown inconsistent and potentially unsustained effects. Specifically for the mdHACM material, a Phase 2B clinical trial was completed by MiMedx Group, Inc. in 2021 to assess efficacy of a single injection in patients with knee OA, with two primary efficacy endpoints (Visual Analog Scale (VAS) for Pain and Western Ontario and McMaster Universities (WOMAC) Osteoarthritis index). The study showed mixed results as the treatment group in one patient cohort demonstrated statistically significant improvement over placebo, whereas the second patient cohort and total patient cohort showed a positive response to both mdHACM and placebo with no significant difference between the groups (MiMedx Group and Inc, 2021). Results such as these highlight some of the complexities and potential sources of variability in clinical studies evaluating candidate therapeutics for effects on structural, pain, and functional aspects of knee OA.

One factor to consider in evaluating therapeutic efficacy is the timing of delivery as it relates to the degenerative state of joint tissues at the time treatment is administered, or in other words, how much the disease has already progressed by the time of delivery. A preclinical study by Raines et al., where MMT animals were treated 2 weeks after surgery with a particulate amniotic membrane/umbilical cord combination product, assessed disease progression at one and 4 weeks after treatment. The study showed potential dose- and time-dependent changes in disease progression metrics at 3 and 6 weeks post-surgery, or 1 and 4 weeks post-injection (Raines et al., 2016). Authors of this work delivered treatment at different post-surgery timepoints and assessed joint tissue changes at different post-treatment timepoints compared to our study, yet they also showed potential chondroprotective effects particularly at earlier post-injection timepoints for the higher dose. These preclinical results coupled with the mixed clinical study results suggest that therapeutic efficacy may depend on timing and disease progression for each joint tissue at the time of treatment.

Other factors to consider when attempting to understand the causes of variability in therapeutic efficacy include questions around donor differences, specificity of the *in vitro* assays used in characterizing potency/activity, changes in product manufacturing processes over time, and potency loss that might occur on the shelf as time passes. Two notable previous studies emphasize concerns that arise around donor-to-donor variability. Our work with mdHACM in this study utilized single donor material. Some studies have utilized pooled donor material as an approach to eliminate effects of donor variability. In one such study Reece et al., 2020, different formulations of mdHACM with varying particle size distributions were used for therapeutic assessment. One of the formulations was the traditional particle size of mdHACM–same size distribution used for this study as well as the prior Willett et al., 2014 shorter-term study (Thote, 2014). The Reece study (Reece et al., 2020) used pooled particulated material from 5 donors compared to use of single donor material in the Willett study (Willett et al., 2014; Thote, 2014). Contrary to Willett study, the Reece study showed that mdHACM did not reduce proteoglycan loss or cartilage tissue swelling in the medial third of the medial tibial plateau compared to saline treatment. There are several possible explanations for the differences in results. The single donor material in the Willett study could have been more potent for chondroprotection than the pooled material in the Reece study. Higher lesion volumes in the Reece study may have contributed to higher joint degeneration severity that could not be overcome by the therapeutic effects of mdHACM. These results taken together with our study’s findings and the mixed results of the recent Phase 2B clinical trial illustrate that variability in outcomes can be caused by several possible factors that must be acknowledged and further explored.

In this study, we highlighted the importance of utilizing quantitative 3D µCT to comprehensively assess therapeutic efficacy in a disease that impacts all the tissues of the joint. A cautionary recent study by McKinney et al. indicated that while an encapsulated mesenchymal stem cell-based therapy provided chondroprotection in the rat MMT model of established OA (with the same delivery timing as this study), the therapy had no effect on subchondral bone changes and exacerbated osteophyte changes compared to saline controls (McKinney et al., 2019). While osteophyte development has been considered as highly associated with cartilage degradation in OA, degenerative changes for each tissue may manifest or be sequenced differently for different individuals (van denBerg, 1999; van der Kraan and van den Berg, 2007). This highlights the importance in preclinical OA research of reporting treatment timing and quantitative metrics for each joint tissue type and is aligned with recent work suggesting that specific pathophysiology and OA phenotype may play a role in treatment efficacy for patients (Mobasheri and Batt, 2016; Roze et al., 2016; Salazar-Noratto et al., 2019a; Van Spil et al., 2019). The Raines study and our study complement each other and independently suggest that amniotic membrane ECM-based particulate treatments could provide therapeutic benefit for cartilage, subchondral bone, and osteophyte tissues with a single injection in joints that are already experiencing osteoarthritic degeneration. However, more work to understand effect mechanisms, therapeutic timing, and causes of therapeutic variability for various placentally-derived materials in osteoarthritic environments is still needed.

While the mechanistic processes by which mdHACM may produce a therapeutic effect are not clearly understood, one contributing factor may be that mdHACM contains hundreds of factors, including basic fibroblast growth factor (bFGF), transforming growth factor-β (TGF-β), and tissue inhibitors of metalloproteinases (TIMPs) (Koob et al., 2014a; Koob et al., 2013; Koob et al., 2014b; Koob et al., 2015; Lei et al., 2017). Osteophyte formation results from endochondral ossification brought about by alterations in bone turnover (Gelse et al., 2003; Karsdal et al., 2014) and can be attenuated by bFGF (Sakano et al., 2002). FGF protein signaling, among others, has also been shown to determine the rate of chondrocyte proliferation (Goldring et al., 2006). TGF-β has been implicated in proliferation of perichondrium derived chondroprogenitor cells (Dounchis et al., 1997). TIMPs and other metalloproteinase inhibitors have been implicated in reducing subchondral bone sclerosis and cartilage damage in other osteoarthritis models (de Bri et al., 1998). Other potential mechanisms of action for mdHACM may involve its potential as a cell recruiter or cell substrate (Koob et al., 2013; Jin et al., 2007). Additionally, important previous work has provided further understanding of localized responses of joint tissues in the rat MMT model after intra-articular injection of mdHACM via gene expression analyses of harvested tissues from different sites within the joint (Salazar-Noratto et al., 2019a; Salazar-Noratto et al., 2019b). Results of this work demonstrated that pro- and anti-inflammatory markers were upregulated in the medial synovial membrane due to treatment with mdHACM and suggested that immunomodulatory effects of mdHACM may help produce protective effects in the joint microenvironment through synovial membrane mediated processes. Further research toward more fully understanding the mechanisms of action of mdHACM are needed, as the complex composition of mdHACM could mean it would impact different PTOA disease microenvironments differently. One such future direction could be to develop suitable high throughput *in vitro* evaluation methods, such as co-culture or functional organoid models, to examine cellular and genetic responses when different cell types are exposed to mdHACM (Reece et al., 2020).

The use of micronized amnion ECM as a disease modifying treatment is a growing area of investigation in OA research. In this study, comprehensive 3D contrast enhanced μCT was utilized to evaluate the effects of mdHACM on joint tissues in a preclinical post-traumatic OA model. Acute treatment, which was shown previously in a shorter-term study to provide therapeutic benefit, was not shown to protect joint tissues out to a longer-term 6 week endpoint, but a single intra-articular injection of mdHACM at 3 weeks post MMT surgery, in an already osteoarthritic joint, slowed degenerative processes for articular cartilage, subchondral bone, and marginal osteophytes. This study supports the suitability of mdHACM as a clinical intervention, after symptom onset, for OA phenotypes where changes in articular cartilage, subchondral bone, and marginal osteophytes contribute to loss of function.

## Data Availability

The original contributions presented in the study are included in the article/supplementary material, further inquiries can be directed to the corresponding author.
